# Mandibular Canine Transmigration and Its Association With Other Dental Anomalies

**DOI:** 10.1155/ijod/4396434

**Published:** 2026-09-14

**Authors:** Dana Kloudová, Lucie Řehořová, Bohumila Straková, Lucie Šimůnková Vítová

**Affiliations:** ^1^ 2nd Medical Faculty, Charles University, Prague, Czech Republic, cuni.cz; ^2^ Private Firm, Hýskov, Czech Republic

**Keywords:** association, canine transmigration, dental anomalies, mandibular canine

## Abstract

**Purpose:**

To investigate whether there exist any association between mandibular canine transmigration and presence of other dental anomalies.

**Methods:**

Initially, 4000 orthopantomograms (OPGs) from the Department of Dentistry, 2nd Faculty of Medicine, Charles University in Prague and one private practice between 2010 and 2024 were evaluated for the presence of mandibular canine transmigration. A total of 120 patients with mandibular canine transmigration were selected. Afterwards, 25 patients with odontoma, other tumours, fibrous dysplasia, craniofacial syndromes, cysts and trauma were removed from research, so the final size of the study group in this retrospective study was 95 patients with mandibular canine transmigration. The control group consisted of 99 patients with physiologically erupted mandibular canine. On CBCT, presence of 16 dental anomalies was evaluated: agenesis of maxillary lateral incisor, mandibular incisor, maxillary and mandibular second premolar, impaction of other teeth, multiple impaction, transposition, transmigration of other teeth, infraocclusion of deciduous molars, distoangulation of mandibular second premolars, taurodontism, rotation, microdontia of maxillary lateral incisor and second premolar and peg‐shaped lateral incisor.

**Results:**

About 95 patients with mandibular canine transmigration were analysed (48 males, 47 females). No statistically significant difference was found for any dental anomaly (*p* ≥ 0.05).

**Conclusions:**

Association between mandibular canine transmigration and other dental anomalies was rejected.

## 1. Introduction

Tooth impaction is a common dental anomaly that occurs when a tooth fails to erupt into the oral cavity in its physiological time, is stuck in the alveolar bone and probably has exhausted its eruption force [1, 2]. Root has finished its development, and the delay of eruption is usually more than a year [3]. Except for third permanent molars, the most frequent impacted teeth dentists and orthodontists can meet with in their practice are permanent maxillary canines [4]. Their prevalence in the general population is in the range between 0.29% and 4.7%, although there are some differences for populations [5–9]. Vice versa, impaction of permanent mandibular canines is very rare. Their incidence was calculated approximately 20 times lower than by maxillary canines [10]. Another statistical research stated that from all diagnosed 858 impacted teeth, there were 91 impacted maxillary canines (10.6%) and only 4 impacted mandibular canines, which is only 0.46% of all impacted teeth and 23 times lower incidence than by maxillary canines [2]. According to population, race and methodologies of measurements, in other studies, there is an incidence of mandibular canine impaction in the range of 0.3%–2.8%, although some studies measured incidence up to 5.1% [11–15]. A common phenomenon associated with mandibular canine impaction is transmigration, when at least half of crown of impacted canines crosses the midline. This modern definition comes from Javid [16] and Joshi [17] modified it as a tendency of canine to cross the midline, as this dental anomaly can be diagnosed earlier when the tooth only horizontalized its position but has not crossed the midline yet. It is believed it commences at an early stage of mixed dentition (6–8 years) [18, 19]; therefore, authors of these studies recommend doing orthopantomogram (OPG) at this age during regular checkup. Their incidence is even lower, which ranges from 0.10% to 0.31% [20, 21]. Earlier, it was believed that transmigration is associated exclusively only with mandibular canines, but newer studies confirmed its occurrence also by maxillary canines [22, 23]. The mechanism of impaction is generally unknown, although many studies paid attention to this problematic, especially for maxillary canines. Two main theories have been established: the guidance theory and genetic theory. According to the guidance theory, represented mainly by Becker and Chaushu´s studies [24–26], root of maxillary lateral incisor fulfils functions of guidance for erupting canine. If development of root is delayed, root is too short or has an altered shape, guidance for canine is insufficient and canines becomes impacted. The genetic theory sees the cause of impaction strictly in the genetic factors, in genes mutation, respectively [27, 28]. Not much have been written about mandibular canine impaction. Dalessandri et al. [29] in their systematic review found only 13 studies. Local factors as aetiology are clear: odontomas, supernumerary teeth, cysts and crowding [30]. Yet there remains still large amount of impacted mandibular canines which aetiology is unknown, majority of them are transmigrated. Some authors claim mandibular canine impaction have strong genetic determinants, as this dental anomaly is often accompanied by other dental anomalies [12, 31].

In previous literature, there are not many studies mentioning associated dental anomalies, as most of this small number of studies dealing with mandibular canine transmigration are primarily case studies [19, 32, 33]. There are only a few retrospective studies aimed at the association between mandibular canine impaction and other dental anomalies, but sample size comprised from 32 mandibular canines only [34]. To our best knowledge, this study has the biggest sample size up to now with the aim to investigate whether there exist any association between mandibular canine transmigration and the presence of other dental anomalies, which could help dentists or orthodontists with early diagnosis and to contribute to make the treatment easier, because mandibular canine transmigration is always a challenge for orthodontic treatment.

## 2. Methods

**Figure 1:**
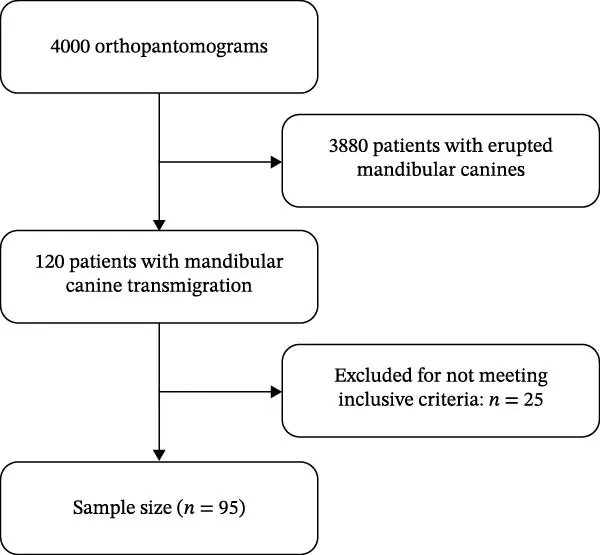
Flowchart of participants in the study group.

The original sample size of this retrospective study comprised of 4000 patients from the Department of Dentistry, 2^nd^ Faculty of Medicine, Charles University in Prague and one private practice between 2010 and 2024. All these patients had proper conventional pretreatment records, including OPGs (Gendex GXDP‐700, KAVO). On the basis of analysis of OPGs provided by three workers (Dana Kloudová, Lucie Šimůnková Vítová and Bohumila Straková), 120 patients with mandibular canine transmigration were selected. All these mandibular canine transmigrations were confirmed on cone beam computer tomography (CBCT; i‐CAT 17–19, Imaging Science International, Hatfield, PA, USA) and subsequently surgically. Inclusion criteria for embodiment into this study were healthy patients with at least one mandibular canine transmigration, no previous orthodontic treatment, good quality of pretreatment records and no trauma in history. Exclusion criteria were craniofacial syndromes, odontomas, other tumours, fibrous dysplasia, cysts, trauma and radiographs with poor quality. Patients with these diagnoses were excluded so there stayed only patients with unknown aetiology, which could indicate some genetic factor. Afterwards, 25 patients were removed from the research because of these selection criteria. Finally, 95 patients (48 males, 47 females) with a mean age of 11.4 ± 1.3 years were included into the study group (Figure 1). All transmigrated in–out study group were impacted, there was no transmigrated canine. The control group consisted of 99 patients with both physiologically erupted mandibular canines. All these patients underwent orthodontic treatment and were of a similar age and gender like patients from study group (54 males, 45 females, mean age 11.5 ± 1.3 years). No CBCT was made for the purpose of this study. This study obtained approval from Ethics Committee of 2^nd^ Faculty of Medicine, Charles University in Prague and meets the tenets of the Declaration of Helsinki.

The study group and control group were examined for the presence of 16 dental anomalies.

Tooth agenesis is a dental anomaly when there is a developmental absence of a tooth in the primary or permanent dentition [35]. Four types of tooth agenesis were evaluated as follows:

1. Agenesis of maxillary lateral incisor.
2. Agenesis of maxillary second premolars.
3. Agenesis of mandibular second premolars.
4. Agnesis of mandibular incisor.
5. Impaction of other teeth is defined as a dental anomaly that occurs when a tooth fails to erupt into oral cavity during their physiological time of eruption [1]. All impacted teeth were included into this group.
6. Multiple impaction occurs when there are two or more other impacted teeth in the dentition [36].
7. Transposition is a dental anomaly that occurs when there is an exchange in the position between two adjacent teeth [37].
8. Transmigation of other teeth occurs when at least half of crown of impacted tooth crosses the midline [16]. This group involves all other transmigrated teeth.
9. Supernumerary teeth are defined as teeth that are in addition to the normal series of deciduous or permanent teeth, either as a single tooth or multiple teeth [38].
10. Infraocclusion of deciduous molars occurs when a deciduous molar is positioned below occlusal plane relative to the adjacent teeth [39].
11. Microdontia of maxillary lateral incisor is a dental anomaly characterised by a decreased size of a crown or the whole tooth, usually in all dimensions. The difference from common diameter is more than 2SD [40].
12. Microdontia of second premolars can be defined similarly as those in lateral incisor—teeth are smaller than normal. They have a smaller volume, too [41].
13. Peg‐shaped maxillary lateral incisor is a dental anomaly when a tooth has an altered shape of crown. The diameter narrows from the cervix to the incisal edge [28].
14. Distoangulation of second premolar is a dental anomaly that occurs when a crown is angulated distally; angle between long axis of the tooth and a line perpendicular to the occlusal plane is more than 15° [42].
15. Tooth rotation occurs when a tooth turns around its long axis [43].
16. Taurodontism is a dental anomaly defined as a developmental disturbance when the tooth has an enlarged pulp chamber and apical displacement of the pulpal floor. The crown is enlarged, the roots are significantly shorter [44].

## 3. Statistical Analysis

IBM SPSS Software for Windows (Version 24.0, IBM Corp, NY, USA) was used for statistical analysis. To determine the minimum sample size, an a priori power analysis using G^∗^Power was selected. It showed that the minimum needed number of subjects in the study is 81 (power = 0.81; *α* = 0.05; and effect size = 0.29). The level for statistical significance was predetermined to be lower or equal to 0.05. The frequencies of dental anomalies in patients with mandibular canine impaction were compared with the control group using Fisher´s exact test. This test was also used to calculate the statistical significance in the prevalence of total dental anomalies by patients between the study group and the control group. The *p*‐values were then false discovery rate (FDR) corrected. Confidence intervals (CI = 95%) and the odds ratio (OR) were also calculated. To analyse the reproducibility of diagnoses, re‐examining for every dental anomaly was repeated by the three authors (Dana Kloudová, Bohumila Straková and Lucie Šimůnková Vítová) within 1 month. To evaluate inter‐class reliability, an intraclass correlation coefficient (ICC), a two‐way mixed model was calculated. It showed excellent consistency for all variables (0.91–0.97). For intraclass measurements, one‐way random model was used. Results confirmed high reliability for all variables (0.92–0.99).

## 4. Results

In the present study, from an initial sample of 4000 patients with proper OPGs, 95 patients were diagnosed with at least one transmigrated mandibular canine. All these patients had CBCT consequently. Males:females ratio is almost equal as the study group consisted of 50.5% males (mean age 11.5 ± 1.4) and 49.5% females (mean age 11.4 ± 1.3) for the whole group (Table 1).

**Table 1:** Demographics of patients with mandibular canine impaction and control group.

	Sex	*N*	Age (year) mean + SD
Study group	Male	48	11.5 ± 1.4
	Female	47	11.3 ± 1.2
Control group	Male	54	11.8 ± 1.5
	Female	45	11.2 ± 1.3

Prevalence and distribution of 16 dental anomalies, OR, confidence interval and *p*‐value are presented in Tables 2 and 3. Statistical tests confirmed that there is no statistically important association between mandibular canine transmigration and other dental anomaly as *p*‐value was never lower or equal to 0.05.

**Table 2:** Distribution of dental anomalies in patients with mandibular canine impaction and control group.

Dental anomalies	Study group patients with anomaly	(%)	Control group patients with anomaly	(%)
Agenesis of maxillary lateral incisor	1	1.05	0	0
Agenesis of maxillary second premolar	1	1.05	2	2.02
Agenesis of mandibular incisor	2	2.1	1	1.01
Agenesis of mandibular second premolar	3	3.15	3	3.03
Peg shaped maxillary lateral incisor	1	1.05	1	1.01
Microdontia of maxillary lateral incisor	1	1.05	0	0
Microdontia of second premolar	1	1.05	0	0
Impaction of other tooth	6	6.31	6	6.06
Multiple impaction	1	1.05	2	2.02
Transposition	0	0	1	1.01
Transmigration of other teeth	0	0	1	1.01
Supernumerary teeth	1	1.05	2	2.02
Infraoclusion of deciduous molars	4	4.21	4	4.04
Distoangulation of mandibular second premolars	1	1.05	0	0
Taurodontism	0	0	1	1.01
Tooth rotation	1	1.05	4	4.04

**Table 3:** Dental anomalies, *p*‐value and other statistics.

Dental anomalies	*P*‐correct	OR	CI	*p*‐Value
Agenesis of maxillary lateral incisor	0.79	0	(0; 37.42)	0.49
Agenesis of maxillary second premolar	1	1.932	(0.10; 115.46)	1
Agenesis of mandibular incisor	1	0.476	(0.01; 9.29)	1
Agenesis of mandibular second premolar	0.9	0.959	(0.13; 7.342)	0.62
Peg shaped maxillary lateral incisor	1	0.959	(0.01; 76.04)	1
Microdontia of maxillary lateral incisor	0.79	0	(0; 37.42)	0.49
Microdontia of second premolar	0.79	0	(0; 37.42)	0.49
Impaction of other tooth	0.79	0.957	(0.25; 3.73)	0.12
Multiple impaction	1	1.932	(0.1; 115.46)	1
Transposition	0.79	0	(0; Inf)	0.49
Transmigration of other teeth	0.79	0	(0; Inf)	0.49
Supernumerary teeth	1	1.932	(0.1; 115.46)	1
Infraoclusion of deciduous molars	0.79	0.958	(0.17; 5.31)	0.37
Distoangulation of mandibular second premolars	0.79	0	(0; Inf)	0.49
Taurodontism	0.79	0	(0; Inf)	0.49
Tooth rotation	0.79	3.933	(0.38; 196.76)	0.37

Abbreviations: CI, confidence interval; OR, odds ratio.

The most frequent dental anomaly in study group was impaction of other teeth: prevalence rate is 6.32% (6 patients). The same prevalence occurred in control group (6 patients). The second most frequent dental anomaly in patients with mandibular canine transmigration was infraocclusion of deciduous molars (4 patients, 4.21%), the same frequency occurred again in control group (4 patients, 4.04%). Three patients with mandibular canine transmigration had agenesis of mandibular canine impaction, one patient was diagnosed with agenesis of mandibular incisor and another one patient with agenesis of maxillary lateral incisor. A very similar frequency of teeth agenesis occurred in control group. There was only one patient in study group who had both impaction of other tooth and agenesis of mandibular second premolar. In control group, no patient had two or more other dental anomalies simultaneously.

Peg‐shaped maxillary lateral incisors showed no difference between patients with mandibular canine impaction and control group (1 patient in both groups). Microdontia (of second premolar and maxillary lateral incisor) occurred only in control group.

Transposition, transmigration of other teeth and taurodontism occurred only in control group (1.01%), distoangulation of mandibular second premolar occurred only in study group (1.05%).

The patient‐level comparison (Table 4) revealed that the overall prevalence of dental anomalies did not significantly differ between the study and control groups (*p* = 0.64). This indicates that patients in the study group do not have a higher clinical predisposition to developing at least one other dental anomaly compared to the control group.

**Table 4:** Patient‐level prevalence of dental anomalies: comparison between groups.

Group	Patients with anomalies	Patients with no anomalies	Total	Prevalence (%)	Fisher´s exact test
Study group	23	72	95	24	0.64
Control group	28	71	99	28	

## 5. Discussion

This study focused on the discovery of any association between mandibular canine transmigration and other dental anomalies. It consisted of 95 patients with mandibular canine transmigration, which is according to our knowledge one of the biggest sample size up to now.

Diagnosis of mandibular canine transmigration is easy and often coincidental during routine checkup by general dentists on an OPG, as it is usually asymptomatic [30, 45]. Only in a rare case is canine transmigration diagnosed because patients seek dentist due to pain, sensitivity or absence of permanent canine and persistence of its predecessor [16]. This could be considered as disadvantageous, because diagnosis is often set too late to prevent from progressing of the condition. The presence of other dental anomalies could alert general dentists and orthodontists of the presence of higher probability of mandibular canine transmigration and should enable to solve the problem sooner.

In maxillary canine impaction, association between maxillary canine impaction and peg‐shaped lateral incisors has been well documented. For example, Becker et al. [46] found in his study 16% patients with peg‐shaped lateral incisors and maxillary canine impaction (Stellzig et al. [47] even in 35%). Sacerdoti and Baccetti [48] found association with microdontia in 17% patients (Marra et al. [49] in 35%). Some studies confirmed association with peg‐shaped maxillary lateral incisor and agenesis of lateral incisor [50], some authors confirmed association with peg‐shaped maxillary lateral incisor, agenesis of lateral incisor, transposition and other tooth impaction [51].

In this study, a relatively high number of patients with mandibular canine transmigration were analysed, 95. Sajnani et al. [52] analysed 64 patients, Guarnieri et al. [21] 51 patients, but none of them analysed association with other dental anomalies. In our study, no statistically important difference has been confirmed for any dental anomaly between patients with mandibular canine transmigration and control group. The most frequent dental anomaly in patients with mandibular canine transmigration in our study was impaction of other teeth (5 maxillary canines, 1 central incisor), but the same frequency was also in the control group. Transposition, transmigration and taurodontism were present only in control group (one patients for every anomaly). Distoangulation of second premolars were not present in control group, only in study group (1 patient).

There are only a few studies analysing association between mandibular canine impaction and other dental anomalies. Shapira et al. [53] analysed 73 patients with mandibular canine impaction and found 9.5% patients with hypodontia. Azeem et al. [54] analysed 20 patients and found no statistical significance between mandibular canine impaction and shape, structural, number or positional dental anomalies. The biggest sample size up to now comprised 93 patients [55], focus was on dentoskeletal features primarily. In this retrospective study of 93 Polish patients with mandibular canine transmigration only three other dental anomalies were found during clinical examination: peg‐shaped maxillary lateral incisor, agenesis of maxillary lateral incisor and mandibular premolars, but similarly as in our study, there was found no statistically important difference between study and control group. Very surprising results brought the newest study analysing the association between mandibular canine impaction and other dental anomalies: in 32 patients were confirmed statistical importance in mandibular canine impaction, tooth impaction, transposition and transmigration [34]. To our best knowledge, there is no study analysing patient‐level comparison of overall prevalence of dental anomalies between the study and control groups. In our results, there was no statistically important difference. The disadvantage of this study is a relatively small sample size, so results could have been influenced by coincidence. AI or other methods could avoid misinterpreting or omitting dental anomalies.

Tooth impaction is always a complication that prolongs the treatment time. Therefore, it is important to diagnose it as soon as possible. As eruption of mandibular canine takes place sooner in early mixed dentition and there is no association between mandibular canine transmigration and presence of other dental anomalies, it is important to make OPGs at the age of 7–8 years that enables analysis of eruption path of the tooth eventually to measure the angle between long axis of the tooth and the midline. Early intervention can prevent from complication and prolongation of orthodontic treatment.

## 6. Conclusion

This retrospective study confirmed that there is no statistically important association between mandibular canine transmigration and any dental anomalies. As treatment of mandibular canine transmigration is always difficult and prolonged, it is important to set the diagnosis in time. As general dentists, by regular checkup have no hints in the presence of other dental anomalies, OPGs in early age remains crucial in early capture of mandibular canine impaction.

## Author Contributions

Dana Kloudová created the material preparation. Dana Kloudová performed data collection. Dana Kloudová and Lucie Řehořová performed analysis. Dana Kloudová, Lucie Šimůnková Vítová, Lucie Řehořová and Bohumila Straková wrote the first draft of the manuscript. All the authors commented on versions of the manuscript and also contributed to the study conception and design.

## Funding

This study received no funding, grants, or other support.

## Disclosure

All authors have read and approved the final version of the manuscript. Corresponding author had full access to all of the data in this study and takes complete responsibility for the integrity of the data and the accuracy of the data analysis.

## Ethics Statement

This study obtained approval from the Ethics Committee of 2^nd^ Faculty of Medicine, Charles University in Prague and meets the tenets of the Declaration of Helsinki.

## Consent

Consent was not required as there were only used anonymized RTG or CBCT retrospectively.

## Conflicts of Interest

The authors declare no conflicts of interest.

## Data Availability

The data supporting this research are available from the corresponding author upon reasonable request.
